# Isolation of rhizosheath and analysis of microbial community structure around roots of *Stipa grandis*

**DOI:** 10.1038/s41598-022-06708-4

**Published:** 2022-02-17

**Authors:** Ai-Min Zhu, Qian Wu, Hai-Li Liu, Hai-Lian Sun, Guo-Dong Han

**Affiliations:** 1 College of Grassland, Resources and Environment, Key Laboratory of Grassland Resources of the Ministry of Education of China, Key Laboratory of Forage Cultivation, Processing and Higher Efficient Utilization of the Ministry of Agriculture and Rural Affairs of China, Inner Mongolia Key Laboratory of Grassland Management and Utilization, Inner Mongolia Agricultural University, Hohhot, 010019 China; 2 Research Base of the Academy of Agriculture and Animal Husbandry of Inner Mongolia, Hohhot, 010031 China

**Keywords:** Ecology, Microbiology, Plant sciences

## Abstract

Root zone microbial structure is particularly complex in plants with rhizosheaths, and greater understanding of the rhizosheath may play an important role in the future development of sustainable agricultural practices. However, one important reason to focus study on rhizosheath microbial structure is that there is no definite method for rhizosheath separation. The aim of this study was to explore rhizosheath isolation methods and the diversity characteristics of microorganisms around the rhizosphere. In this study, we isolated the rhizosheath of *Stipa grandis*, a dominant species in desert steppe, and the microorganisms in the roots, root epidermis, rhizosheath and rhizosphere soil were extracted and sequenced by 16S rRNA and ITS. The alpha diversity index of bacteria in *Stipa grandis* rhizosphere soil was the greatest, followed by rhizosheath, and the alpha diversity index of endophytic bacteria in root system was the smallest. The alpha diversity index of fungi in the rhizosheath and rhizosphere soil were significantly higher than that in the root epidermis and root system. There were significant differences in bacterial community structure between the root epidermis, endophytic bacteria, rhizosheath and rhizosphere soil. Unlike bacterial community structure, the community structure of fungi in the root epidermis was similar that of endophytic fungi, but significantly different from those in rhizosheath and rhizosphere soil. This study demonstrated a feasible method for separating plant rhizosheath and root epidermis. We suggest that the root epidermis can act as the interface between the host plant root and the external soil environment. We will have to re-examine the biological and ecological significance of rhizosheath and microorganisms in rhizosheath, as well as the mechanism explaining the close relationship of the rhizosheath and the plant root epidermis. This study provides theoretical and technical guidance for the isolation of the plant rhizosheath and the study of microorganisms in plant rhizosheath.

## Introduction

The close interaction between plants and rhizosphere microorganisms has prompted people to regard plants as a holobiont. Research on plant root and rhizosphere soil microorganisms aims to identify correlations between plants, soil and microorganisms, and reveal their important roles in the ecosystem. However, the relationship between the roots and the soil around the roots of plants with rhizosheaths is complex. For example, barley^1^, wheat^2^, corn^3^ and rushes^4,5^ have rhizosheaths, and some scholars have proposed that there is a rhizosheath on the fine roots of some leguminous plants^6,7^. However, McCully^8^ suggests that this needs further research and confirmation. Although, it has been more than 100 years^9^ since the rhizosheath was first described, initiating study of its structure^10^, formation, function and genetic characteristics, the rhizosheath remains poorly understood.

The rhizosheath is particularly obvious in the root structure of *Gramineae* in arid areas. Price^11^ and Young^12^ suggest that the rhizosheath plays an important role in increasing the drought resistance of plants, and existing research supports this view^13–15^. This understanding is of great significance for improving agricultural sustainability in the context of future climate change, limited resources and a growing global population. Some scholars believe that rhizosheath plants may play an important role in the second green revolution and the future development of sustainable agricultural practices^16,17^.

Research on the structure and function of plant rhizosphere microorganisms has long been a hot topic. Although most microorganisms in the environment have not been cultured, with the rapid development of high-throughput sequencing technology, the structure of microorganisms in the environment is gradually becoming known. Previous studies have shown that there are some differences in the composition of plant root microorganisms and rhizosphere and non-rhizosphere soil microorganisms, and that these differences directly affect the growth of host plants. For example, some high concentrations of molecules released by rhizosphere microorganisms inhibit the elongation of primary roots and promote the formation of lateral roots and root hairs^18^. Some rhizosphere bacteria or fungi produce auxin, which directly interferes with auxin signal transduction^19^. Plant rhizosphere microorganisms play an important role in improving crop yield and resistance. Recent reviews have shown that rhizosphere microorganisms and drought resistant crops interact through several different mechanisms to respond to climate change^18^. Different plant species or genotypes can select different rhizosphere microbial communities by producing different secretions from their roots. Although there is still uncertainty about global climate change, there are signs that global temperature will continue to rise, and that drought frequency and duration will change in many locations. Plants respond to these stresses through self-regulation. A recent review by Vries^20^ found little evidence for a coupling relationship between the drought tolerance mechanism of microorganisms and the functional characteristics of plant drought resistance, highlighting the need for further research. However, there are few reports on the structure and function of microorganisms in plant rhizosheaths. Therefore, it is still a challenge to study microorganisms around the rhizosheath plants.

York et al.^21^ summarized and defined the generation process and semantics of the "rhizosphere", and considered that rhizosheath is a mixture of soil particles adhered by mucus (the secretion of plant roots or microorganisms)^1,9^ and that the rhizosheath is part of the plant rhizosphere. The epidermal cell layer attached to the rhizosheath is not a part of the rhizosheath, and they called the combination of the epidermal cell layer and the rhizosheath a "rhizoplane"^21^. The purpose of our study was to isolate root sheath and root epidermis and to extract microbial DNA from them. The difficulty of rhizosheath separation lies in the separation of the rhizosheath and the root epidermis. There is also no standard method to isolate microorganisms from the rhizosheath. In this study, using *Stipa grandis* as experimental material, we isolated the roots, root epidermis, rhizosheath and rhizosphere soil of *Stipa grandis*, extracted microbial DNA from roots, root epidermis, rhizosheath and rhizosphere soil, and sequenced microorganisms by 16S rRNA and ITS. The purposes of our research were to explore the methods for rhizosheath and root epidermis isolation, and to analyze the similarities and differences between microbial communities in the roots, root epidermis, rhizosheath and rhizosphere soil of *Stipa grandis*, so as to provide new methods and suggestions for future research on rhizosheath plants.

## Materials and methods

### Overview of the research site

The experiment was carried out in Maodeng Pasture (116.03°E–116.50°E, 44.80° N–44.82°N), Xilinhot City, Inner Mongolia, China. The area has a temperate arid continental climate, and lies at an elevation of 1055 m. The annual average temperature is 0–1 °C, the frost-free period is 90–115 d, and the accumulated temperature greater than or equal to 0 °C is 1800 °C.

### Test sample collection

*Stipa grandis*, a typical gramineous plant in desert steppe, is a constructive species. Samples were taken from Maodeng Pasture (*Stipa grandis* steppe) in Xilinhot City, Inner Mongolia, in July 2019. We designed 5 replicates and randomly sampled at 5 sites 50 m apart. Three *Stipa grandis* individuals were collected from each site as one sample of replicates, with each plant excavated to a depth of 20 cm. The mixed samples of plant and soil were put into plastic bags and immediately placed into ice boxes, before being brought back to the laboratory for root, root epidermis, rhizosheath and rhizosphere soil separation. The isolation of roots, root epidermis, rhizosheath and rhizosphere soil and the extraction of microbial DNA were carried out at the Sino-Dutch joint laboratory of the Grassland and Resources College of Inner Mongolia Agricultural University.

### Rhizosphere, rhizosheath, root epidermis and root harvesting

Phosphate buffer is needed before separating plant roots, root epidermis, rhizosheath and rhizosphere soil. Phosphate buffer plays an important role in maintaining microbial activity and does not change the composition of the sampled microorganisms. Phosphate buffer: PB, per litre: 6.33 g NaH_2_PO_4_·H_2_O, 10.96 g Na_2_HPO_4_·2H_2_O and 200 μL Silwet L-77.

**Figure 1:**
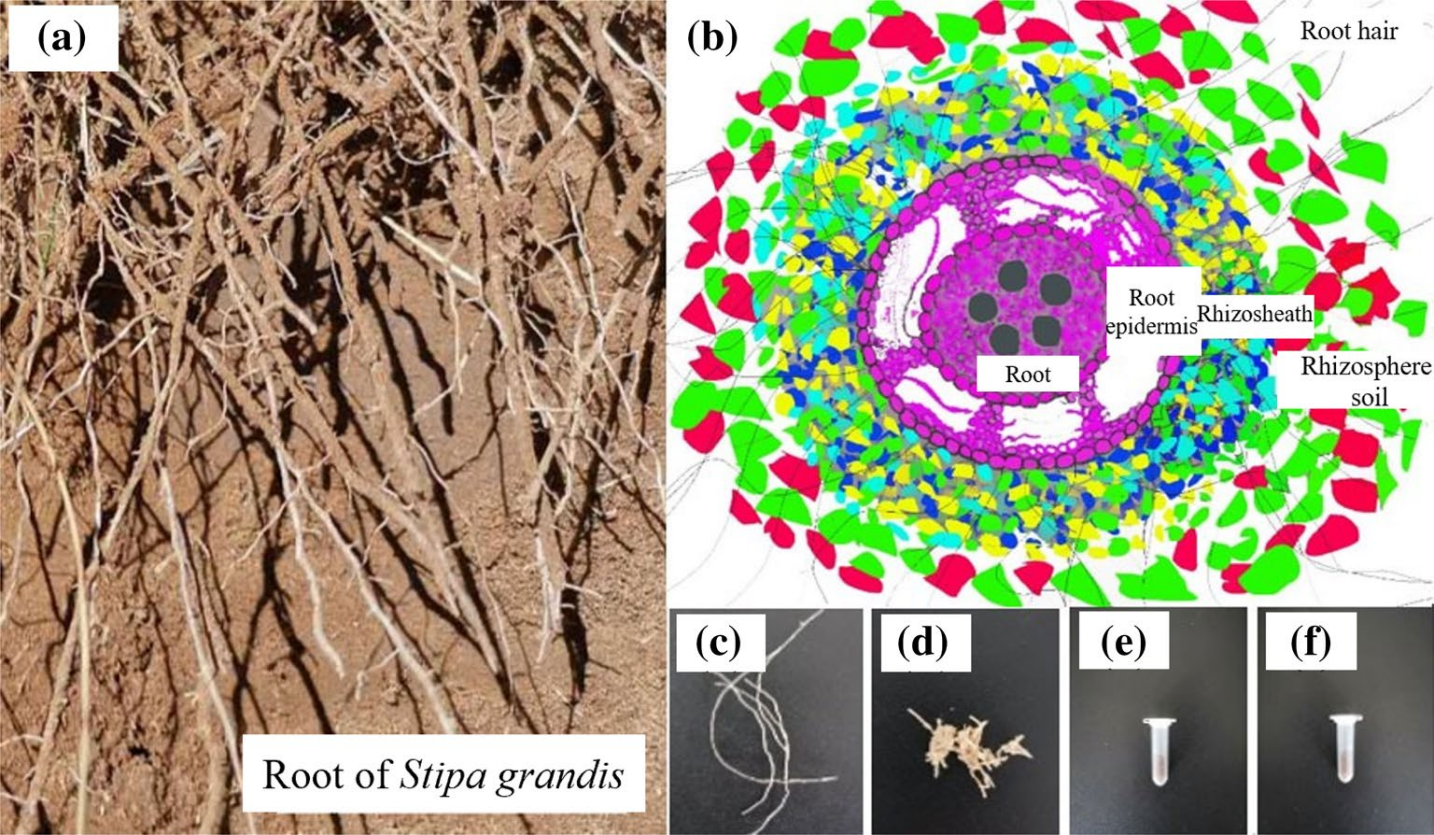
Root system of *Stipa grandis* and schematic diagram of the root cross section. (**a**) *Stipa grandis* roots. (**b**) Schematic diagram of the cross section of the root system, and (**c**), (**d**), (**e**) and (**f**) are the root system, root epidermis, rhizosheath and rhizosphere soil, respectively.

Figure 1a shows the root system of *Stipa grandis*, and a schematic diagram of the cross section of the root system of *Stipa grandis* is shown in Fig. 1b. Starting from the centre and proceeding outwards, the schematic diagram of the cross section of *Stipa grandis* shows the root (Fig. 1c), the root epidermis (Fig. 1d), the rhizosheath (Fig. 1e) and rhizosphere soil (Fig. 1f). The harvesting protocol closely followed procedures described previously^22,23^, with minor modifications, as described here. In Bulgarelli and Lundberg's study, *Arabidopsis thaliana* was taken as the model plant, but the root system of *Arabidopsis thaliana* did not have a rhizosheath structure. Therefore, we made minor modifications to the harvesting method according to the definitions of rhizosheath^1,9,24^ and rhizosphere soil. Sterile gloves were worn and the workspace was sterilized with 70% EtOH. The forceps and scissors used in the experiment were wiped and disinfected with 70% EtOH. We selected 3 *Stipa grandis* plants from each sampling point, removed easily separated large pieces of soil, kneaded and shook the samples with gloved hands, and patted the roots to obtain rhizosphere soil (Fig. 1f). The soil that was not shaken off and that continued to adhere to the root surface was defined as rhizosheath. We placed the root with rhizosheath in a 50 mL tube containing 25 mL phosphate buffer. This was vortexed for max 15 s, and filtered through a 100 μm nylon mesh cell strainer into an empty 50 mL tube. After centrifuging at 4000×*g* for 15 min, we poured out the supernatant. The soil samples left in the 50 ml tube were rhizosheath samples (Fig. 1e). The rhizosphere soil was treated in the same way (Fig. 1f). The root hairs on the filtered root samples were removed with tweezers, and the filtered roots and outer epidermal cell layer carefully separated with tweezers. The root and root epidermis were placed in different 15 ml tubes containing buffer and washed 3 times. The root and outer epidermal cell layer were placed on sterile filter paper and the roots (Fig. 1c) and outer epidermal cell layer sample (Fig. 1d) were air dried for about 6 h.

### Sequencing process (Novogene company)

#### Extraction and PCR amplification of microbial genomic DNA

Microbial DNA from rhizosheath, root epidermis and rhizosphere soil samples was extracted with a MOBIO DNeasy PowerSoil kit 12888-100. Microbial DNA from the roots was extracted with a FastDNATM Spin Kit for Soil^24^. Agarose gel electrophoresis was used to detect the purity and concentration of DNA. An appropriate sample of DNA was applied to the centrifuge tube and diluted with sterile water to 1 ng/ L. Using diluted genomic DNA as a template, according to the selection of the sequencing region, barcoded primers, Phusion® High-Fidelity PCR Master Mix with GC Buffer from New England Biolabs Ltd., and high fidelity enzyme were used for PCR to ensure the efficiency and accuracy of amplification. Using primers 515 F (GTGCCAGCMGCCGCGGTAA) and 806 R (GGACTACHVGGGTWTCTAAT), the V4 region of the 16S gene was sequenced. Using primers ITS5-1737F (CTTGGTCATTTAGAGGAAGTAA) and ITS2-2043R (GCTGCGTTCTTCATCGATGC), the ITS1 region gene was sequenced. The PCR product was detected by electrophoresis with 2% agarose gel. According to the concentration of the PCR product, the samples were mixed equally, and then the PCR products were detected by agarose gel electrophoresis with 2% agarose gel. A gel recovery kit provided by Qiagen company was used to recover the target band. Library construction used a TruSeq® DNA PCR-Free Sample Preparation Kit (Building Database Kit). The library was quantified by qubit and qPCR, and after the library was qualified, it was sequenced by novaseq6000.

### Data analysis

#### Paired-end reads assembly and quality control

Paired-end reads were assigned to samples based on their unique barcodes and truncated by cutting off the barcode and primer sequence. Paired-end reads were merged using FLASH^25^, a very fast and accurate analysis tool, which was designed to merge paired-end reads when at least some of the reads overlap the read generated from the opposite end of the same DNA fragment, and the splicing sequences were called raw tags. Quality filtering on the raw tags was performed under specific filtering conditions to obtain high-quality clean tags^26^ according to the QIIME^27^ quality controlled process. The tags were compared with the reference database using the UCHIME algorithm^28^ to detect chimera sequences, and the chimera sequences were removed^29^ to finally obtain the effective tags.

#### OTU cluster and species annotation

Sequence analysis was performed by Uparse software^29^. Sequences with ≥ 97% similarity were assigned to the same OTUs. Representative sequences for each OTU were screened for further annotation. For each representative sequence, the Silva Database^30,31^ was used based on the Mothur algorithm to annotate taxonomic information. In order to study the phylogenetic relationships of different OTUs, and the differences in dominant species in different samples (groups), multiple sequence alignment was conducted using the MUSCLE software^32^. Information on OTU abundances was normalized by taking the sequence number corresponding to the sample with the least sequences as the standard. Subsequent analysis of alpha diversity and beta diversity were all performed based on the resulting normalized data.

#### Alpha diversity

Alpha diversity was applied to analyze the complexity of species diversity for each sample through 6 indexes, including Observed species, Chao1, Shannon, Simpson’s, ACE and Good’s coverage. All these indices in our samples were calculated with QIIME (Version 1.7.0) and displayed with R Software (Version 2.15.3). Chao1 and ACE indexes characterize community richness; Shannon and Simpson’s indexes are indicators of community diversity; and Observed species and Good’s coverage indexes are indicators of sequencing depth. One-way analysis of variance (ANOVAs) with Duncan’s test were used to evaluate the difference between the Alpha diversity indexes of the four different components (i.e., roots, root epidermis, rhizosheath and rhizosphere), and *p* < 0.05 was used to indicate significant difference.

#### Beta diversity

Beta diversity analysis was used to evaluate differences between samples in species complexity. Beta diversity on both weighted and unweighted unifrac were calculated by QIIME software (Version 1.9.1). The Unweighted Pair-group Method with Arithmetic Means (UPGMA) clustering was performed as a type of hierarchical clustering method to interpret the distance matrix using average linkage, and was conducted by QIIME software (Version 1.9.1). Non-metric multidimensional scaling (NMDS) method is a data analysis method that simplifies the research objects (samples or variables) in multidimensional space to low dimensional space for positioning, analysis and classification, while retaining the original relationship between objects. In this study, NMDS analysis was based on OTU relative abundance and Bray–Curtis distance. The visual graph of NMDS analysis results can show the distance between groups within the microbial community. Vegan software package in R software was used for NMDS analysis. Analysis of molecular variance (AMOVA) introduces the evolutionary distance between OTUs to measure and calculate the squared differences between haplotypes (or genotypes). The squared differences between all haplotypes form a distance matrix, which is the basic data of AMOVA analysis. AMOVA analysis used the AMOVA function in Mothur software, and *p* < 0.05 was used to indicate significant difference between groups.

### Ethics approval and consent to participate

*Stipa grandis*, the species sampled in this study, is not an endangered species, and sample collection had been approved by director Sun Hailian, head of the Research Base of the Academy of Agriculture and Animal Husbandry of Inner Mongolia, who is also one of our co-authors. The sample collection complied with relevant institutional, national, and international guidelines and legislation.

## Results

### Separation of the rhizosheath of *Stipa grandis*

**Figure 2:**
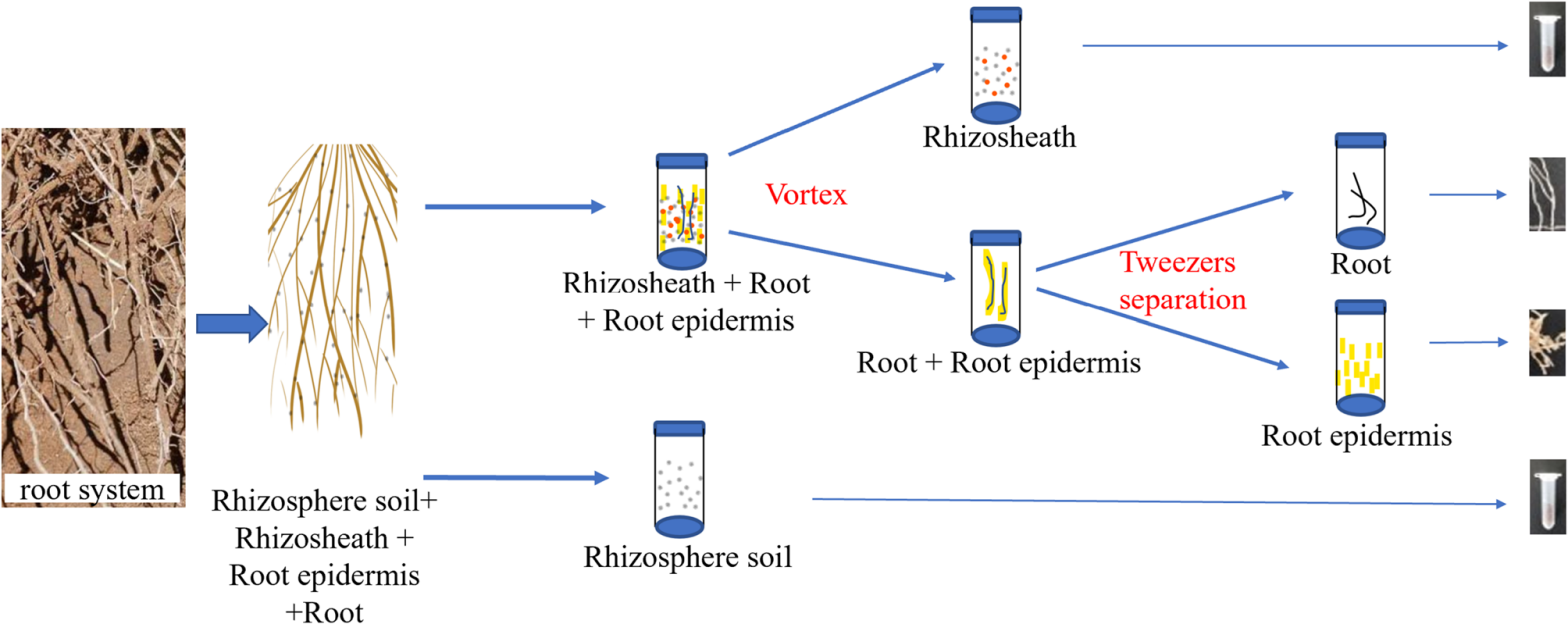
Schematic diagram of the separation process of *Stipa grandis* roots, root epidermis, rhizosheath and rhizosphere soil.

Figure 2 presents a flow chart of the rhizosheath separation process. Using this method, we were able to isolate the rhizosheath, rhizosphere soil, root epidermis and root system of *Stipa grandis*. Firstly, the rhizosphere soil of *Stipa grandis* was collected by shaking the soil. Then, we put the mixture of roots, rhizosheath and root epidermis into a 50 ml centrifuge tube filled with 25 ml phosphoric acid buffer, vortexed and centrifuged the sample, and removed the supernatant to obtain *Stipa grandis* rhizosheath. Then, tweezers were used to separate the root epidermis and the root system. The specific operation is described in detail in the Materials and Methods section.

### Distribution characteristics of bacterial and fungal communities

**Figure 3:**
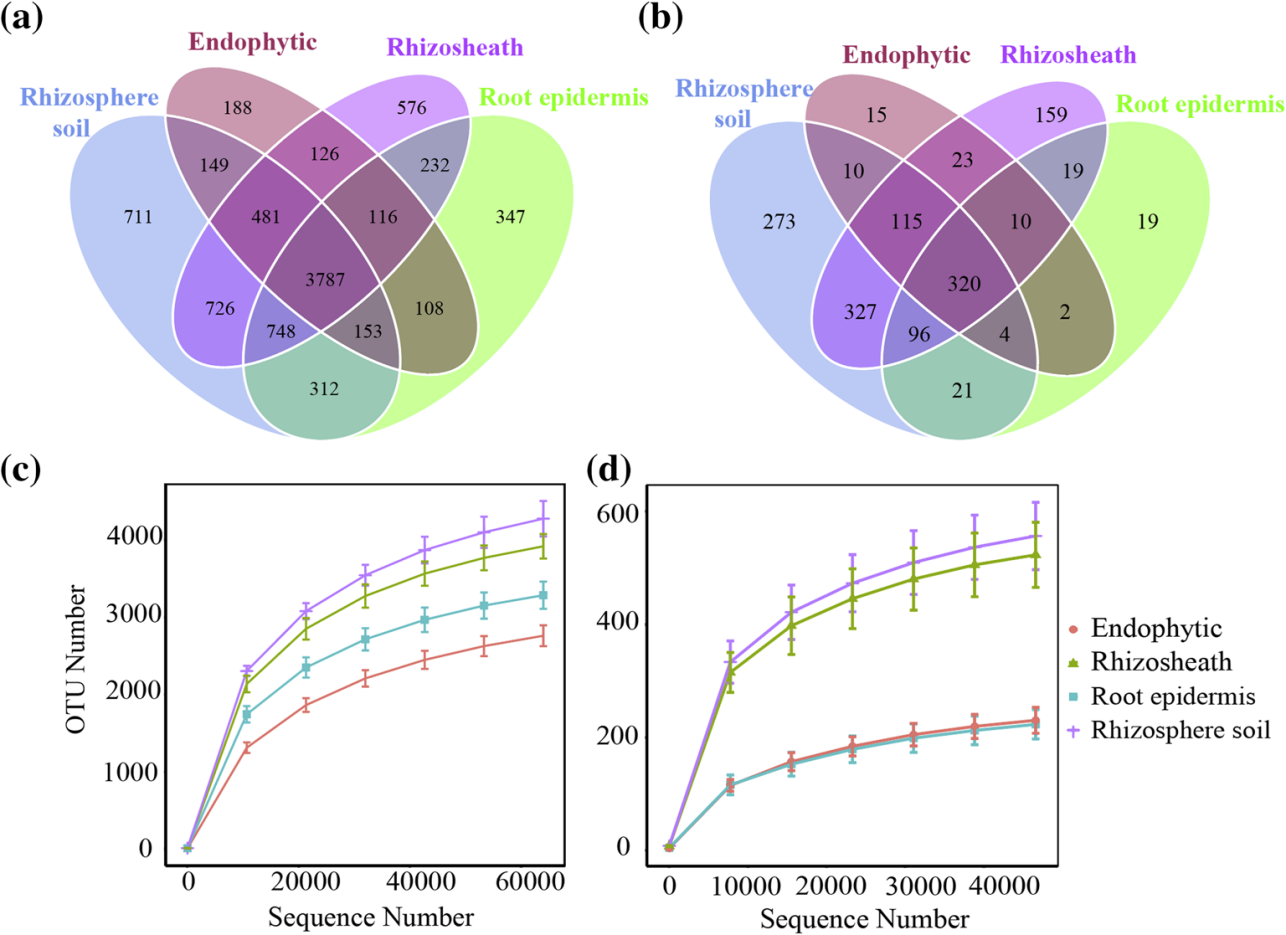
Venn diagrams of samples and rarefaction curves for samples. In (**a**) and (**c**) are bacteria, and (**b**) and (**d**) are fungi. In (**a**, **b**), each circle in the Venn diagram represents a group of samples. The numbers in the overlapping areas represent the number of OTUs shared between groups, and the numbers in areas without overlap represent the number of OTUs unique to the sample group. In (**c**, **d**), the abscissa is the number of sequencing pieces randomly selected from a sample, and the ordinate is the number of OTUs that can be constructed based on the number of sequencing pieces to reflect the sequencing depth.

Bacterial OTUs (Fig. 3a). A total of 8760 OTUs were generated by OTUs clustering at 97% similarity level. Among them, 3787 OTUs were common in roots, root epidermis, rhizosheath, and rhizosphere soil. There were 188, 347, 576 and 711 unique OTUs in roots, root epidermis, rhizosheath, and rhizosphere soil, respectively, accounting for 3.68%, 5.98%, 8.48% and 10.06% of their respective total numbers of OTUs.

Fungal OTUs (Fig. 3b): A total of 1423 OTUs were generated by OTUs clustering at 97% similarity level. Among them, 318 OTUs were common in roots, root epidermis, rhizosheath and rhizosphere soil. The number of unique OTUs in roots, root epidermis, rhizosheath and rhizosphere soil were 11, 19, 164 and 262, respectively, accounting for 2.28%, 3.84%, 15.96% and 22.13% of their respective total numbers of OTUs. The dilution curves of OTUs tended to be gradual, indicating that the measured data could accurately reflect the information on the plant fungal community (Fig. 3c, d).

One-way analysis of variance (ANOVA) with Duncan’s test was used to evaluate the difference between the Alpha diversity indexes of bacterial and fungal communities in the four different components (i.e., roots, root epidermis, rhizosheath and rhizosphere soil), and *p* < 0.05 was used to indicate significant difference. Table 1 shows that the number of Observed species, Chao1 index and ACE index were highest in rhizosphere soil, followed by the rhizosheath, and were smallest in the root system, and there were significant differences between them (*p* < 0.05). There was no significant difference in the Shannon index between rhizosphere soil and the rhizosheath (*p* > 0.05), and the Shannon index for both were significantly higher than for the root epidermis and roots (*p* < 0.05), but the Shannon index for bacteria in root epidermis was significantly higher than in roots (*p* < 0.05). Simpson’s indexes for bacteria in rhizosphere soil, rhizosheath and root epidermis showed no significant differences (*p* > 0.05), but were significantly higher than for the root system (*p* < 0.05). The Good’s coverage index of the samples was higher than 98% (Table 1).

**Table 1:** Alpha diversity of bacteria and fungi in roots, rhizosheath, rhizosphere soil and non-rhizosphere soil.

Microorganism	Sample name	Observed species	Shannon	Simpson’s	Chao1	ACE	Good’s coverage
Bacteria	Endophytic	2769.0 ± 143.9d	5.41 ± 0.31c	0.82 ± 0.03b	3210.4 ± 159.4d	3312.3 ± 174.8d	0.989 ± 0.001
	Root epidermis	3287.4 ± 213.2c	8.87 ± 0.46b	0.99 ± 0.01a	3714.6 ± 248.7c	3798.1 ± 266.4c	0.989 ± 0.001
	Rhizosheath	3919.2 ± 187.9b	9.77 ± 0.28a	1.00 ± 0.00a	4392.5 ± 145.6b	4444.4 ± 156.3b	0.988 ± 0.000
	Rhizosphere soil	4278.6 ± 256.5a	10.10 ± 0.10a	1.00 ± 0.00a	4858.4 ± 512.0a	4938.6 ± 504.8a	0.986 ± 0.003
Fungi	Endophytic	230.2 ± 27.5b	2.38 ± 0.59b	0.62 ± 0.14b	264.3 ± 34.5b	278.1 ± 37.7b	0.999 ± 0.000
	Root epidermis	223.8 ± 29.2b	2.61 ± 0.75b	0.68 ± 0.15b	260.8 ± 33.4b	272.6 ± 31.3b	0.999 ± 0.000
	Rhizosheath	532.2 ± 65.5a	5.11 ± 0.62a	0.89 ± 0.06a	597.7 ± 65.4a	613.4 ± 68.8a	0.998 ± 0.000
	Rhizosphere soil	563.0 ± 70.9a	5.64 ± 0.44a	0.93 ± 0.04a	693.9 ± 163.6a	656.6 ± 83.6a	0.998 ± 0.001

Different lowercase letters in the same column indicate significant differences between bacterial or fungal groups (*p* < 0.05).

The Observed species, Shannon, Simpson’s, Chao1 and ACE indexes of fungi in rhizosphere soil were the highest, followed by the rhizosheath, but there was no significant difference between the two groups (*p* > 0.05). The Observed species, Shannon, Simpson’s, Chao1 and ACE indexes of rhizosphere soil and rhizosheath fungi were significantly higher than those of the root epidermis and root (*p* < 0.05). There were no significant differences in observed species, Shannon, Simpson’s, Chao1 or ACE indexed between roots and root epidermis (*p* > 0.05). The Good’s coverage indexes of the samples were all higher than 99% (Table 1).

**Figure 4:**
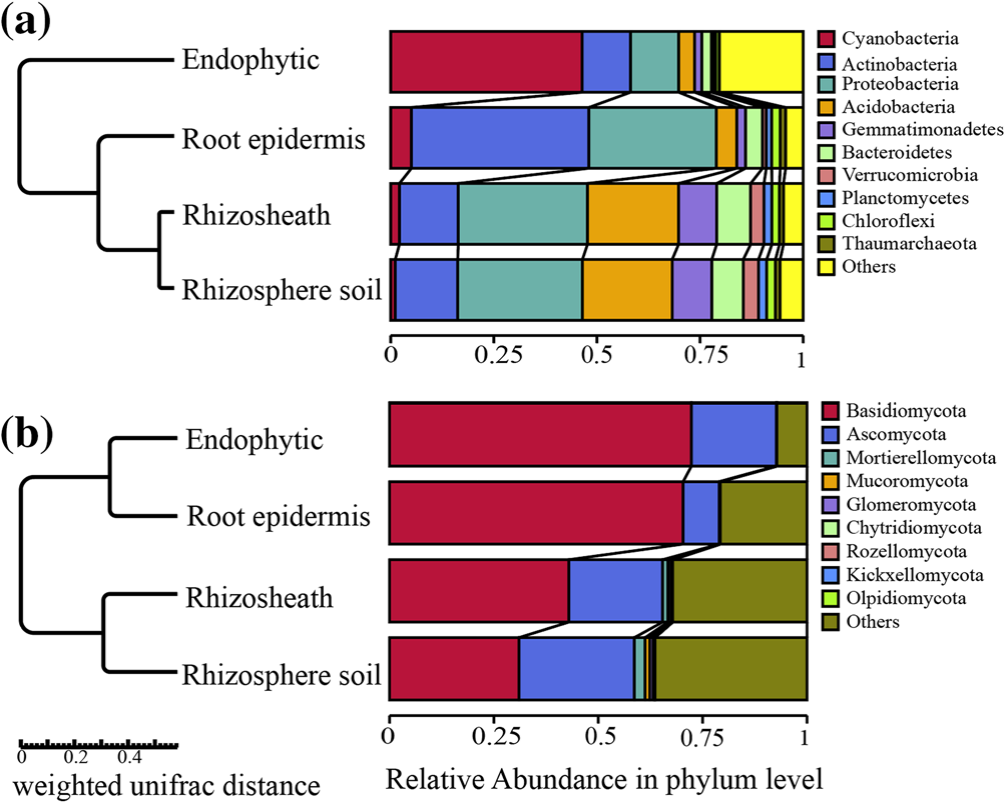
UPMGA clustering tree diagram of species composition at bacteria and fungi phyla level. In (**a**) and (**b**) refer to the UPMGA cluster tree of bacteria and fungi respectively. Both UPMGA cluster trees are based on the relative abundance data of the sample species. The length of the branch represents the distance between samples, and if the community composition of samples is similar, they are clustered into the same cluster in the cluster tree. The relative abundance was the average of 5 replicates.

The top 10 phylum level classification of bacterial communities is shown in Fig. 4a. The relative abundance of *Cyanobacteria* in the root system was 46.4%, while the relative abundances of *Cyanobacteria* in rhizosphere, rhizosphere and non-rhizosphere soil were only 5.0%, 2.1% and 1.1%, respectively. *Actinobacteria* was the dominant population in root epidermis, and its relative abundance was 43.0%, which was higher than that in the root system, rhizosheath and rhizosphere soil. The relative abundances of *Proteobacteria* in root epidermis, rhizosheath and rhizosphere soil were 30.9%, 31.3% and 30.2%, respectively, while that in roots was only 11.7%. The relative abundances of *Acidobacteria*, *Gemmatimonadetes*, *Bacteroidetes* and *Verrucomicrobia* in rhizosheath soil and rhizosphere soil were similar and higher than those in root systems and root epidermis. Their relative abundances in rhizosheath were 22.1%, 9.2%, 8.2% and 3.2%, respectively, and those in rhizosphere soil were 21.8%, 9.6%, 7.6% and 3.7%, respectively.

The top 10 phylum level classification of the sample fungal communities is shown in Fig. 4b. The distribution of fungi abundances among components differed from that of bacterial communities. Analysis showed that *Basidiomycota* and *Ascomycota* had higher relative abundance in roots, root epidermis, rhizosheath and rhizosphere soil. The relative abundances of *Basidiomycota* in roots and root epidermis were 72.3% and 70.3%, respectively, which is higher than in rhizosheath and rhizosphere soil. The relative abundances of *Ascomycota* in roots, rhizosheath and rhizosphere soil were 20.4%, 22.4% and 27.6%, respectively, while that in root epidermis was only 8.7%.

**Figure 5:**
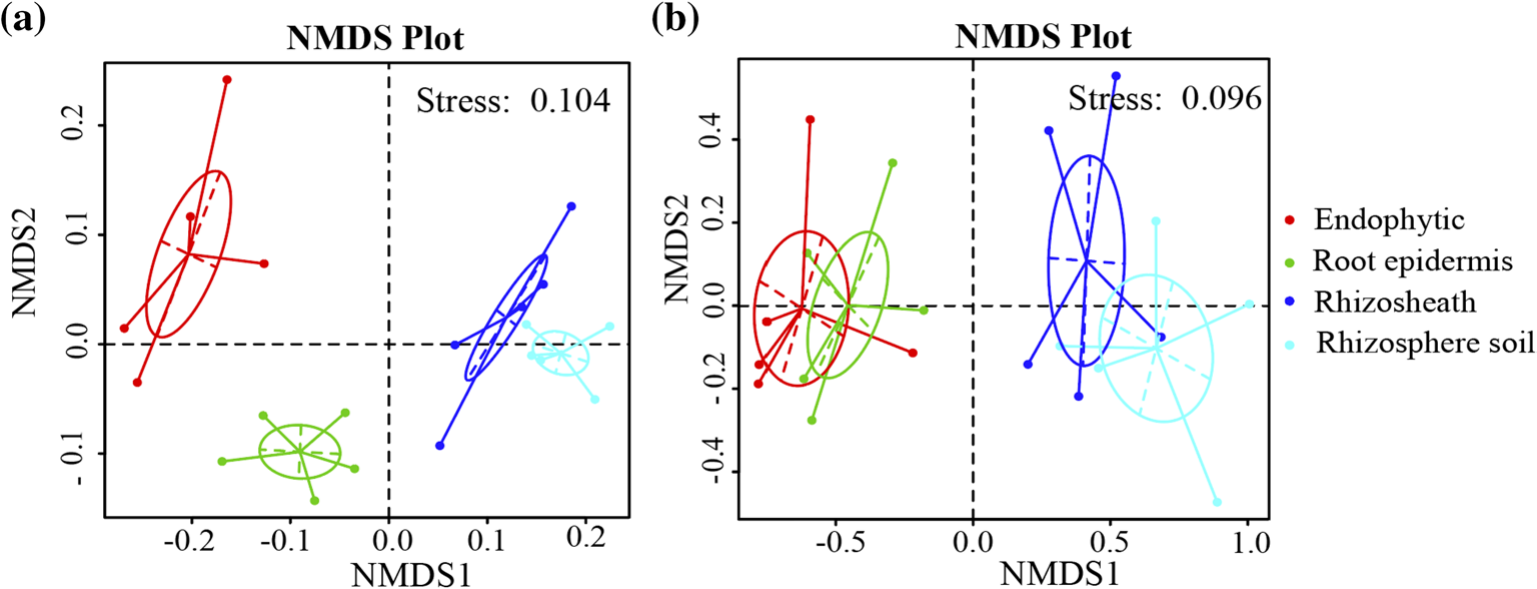
NMDS analysis of bacteria and fungi. In (**a**) and (**b**) refer to the NMDS analysis of bacteria and fungi samples, respectively. Each point in the figure represents a sample. The distance between points indicates the degree of difference, and samples from the same group are represented by the same color and connected by lines and ellipses. When stress is less than 0.2, NMDS can accurately reflect the difference between sample groups.

Results of non-metric multidimensional scaling (NMDS) analysis for bacteria in roots, root epidermis, rhizosheath and rhizosphere soil of *Stipa grandis* are shown in Fig. 5a. The bacterial community structure of root epidermis, endophytes and rhizosphere soil in the NMDS plot is distributed in different regions, indicating that there were differences between them in bacterial community composition, while the bacterial community compositions of rhizosheath and rhizosphere soil were similar.

The results of NMDS analysis for fungi are shown in Fig. 5b. The fungal community structure of roots and root epidermis, and rhizosheath and rhizosphere soil were relatively close, while those of roots and root epidermis were relatively distinct from those of rhizosheath and rhizosphere soil. Through UPGMA cluster analysis of all the samples, in contrast to bacterial community clustering, rhizosheath and rhizosphere soil fungal communities were clustered into one group, while fungal communities in roots and root epidermis were clustered into another group.

As shown in Table 2, the results of analysis of molecular variance (AMOVA) suggest that the bacterial community composition in rhizosheath and rhizosphere soil of *Stipa grandis* were not significantly different (*p* > 0.05). The bacterial community composition of roots and root epidermis of *Stipa grandis* were significantly different at *p* < 0.05, and the differences in bacterial community among other groups were extremely significantly different (*p* < 0.01). There was no significant difference in fungal communities between the roots and root epidermis of *Stipa grandis* or between the rhizosheath and rhizosphere soil (*p* > 0.05). The differences between fungal communities among other groups reached an extremely significant level (*p* < 0.01).

**Table 2:** AMOVA analysis significance test table of difference in bacterial and fungal community structure between groups.

Microorganism	Vs group	SS	df	MS	Fs	p value
Bacteria	Endophytic-Root epidermis -Rhizosheath-Rhizosphere soil	2.18148(0.23445)	3(16)	0.72716(0.01465)	49.6253	< 0.001**
	Endophytic-Root epidermis	1.12673(0.08354)	1(8)	1.12673(0.01044)	107.898	0.013*
	Root epidermis-Rhizosphere soil	0.30559(0.11343)	1(8)	0.30559(0.01418)	21.5525	0.003**
	Rhizosheath-Root epidermis	0.28601(0.15459)	1(8)	0.28601(0.01932)	14.8009	0.005**
	Endophytic-Rhizosheath	1.28529(0.12102)	1(8)	1.28529(0.01513)	84.9651	0.007**
	Rhizosheath-Rhizosphere soil	0.01355(0.15091)	1(8)	0.01355(0.01886)	0.71831	0.672
	Endophytic-Rhizosphere soil	1.34579(0.07986)	1(8)	1.34579(0.00998)	134.82	0.006**
Fungi	Endophytic-Root epidermis -Rhizosheath-Rhizosphere soil	6.63178(6.53519)	3(16)	2.21059(0.40845)	5.41216	< 0.001**
	Endophytic-Root epidermis	0.40740(2.75986)	1(8)	0.40740(0.34498)	1.18094	0.309
	Root epidermis-Rhizosphere soil	3.31283(3.25717)	1(8)	3.31283(0.40715)	8.1367	0.006**
	Rhizosheath-Root epidermis	2.17638(3.47909)	1(8)	2.17638(0.43489)	5.00449	0.002**
	Endophytic-Rhizosheath	2.86233(3.27802)	1(8)	2.86233(0.40975)	6.98551	0.006**
	Rhizosheath-Rhizosphere soil	0.42909(3.77533)	1(8)	0.42909(0.47192)	0.90924	0.541
	Endophytic-Rhizosphere soil	4.07552(3.0561)	1(8)	4.07552(0.38201)	10.6685	0.008**

*Significant difference between groups (*p* < 0.05); **Extremely significant difference between groups (*p* < 0.01). SS stands for total variance, also known as the sum of squares of deviation; df is degrees of freedom; MS is the mean square (difference), i.e. SS / DF; FS is the F test value; p-value is the p value, with a value less than 0.05 indicating significant difference between groups. The values corresponding to the residual items are in brackets.

## Discussion

‘Rhizoplane’ refers to the outer surface of plant roots and any closely attached soil or debris particles, as proposed by Clark^33^. However, some scholars after him have proposed different views, suggesting that only the soil attached to the root can be regarded as the rhizosphere, while the root epidermis washed by soil particles is called the rhizoplane^34–36^. York et al.^21^ suggested that it was incorrect to call root epidermis the rhizoplane, as this would greatly reduce the spatial range of the rhizosphere, and he agreed with Clark's^33^ definition of the rhizoplane. Based on this theory, we separated the rhizosheath, root epidermis and rhizosphere soil of *Stipa grandis*, a species with strong drought resistance in semi-arid desert grassland. Our results showed that there were significant differences in the composition of bacterial and fungal communities in root epidermis and rhizosheath, which could imply that they play different but important roles in plant growth^37^. For example, Fu et al.^38^ showed that endophytes can improve the stress resistance of cotton plants by promoting growth. Studies by van Loon^39^ and Wees^40^ have shown that rhizosphere microorganisms can induce plants to produce broad-spectrum resistance to pathogens. Our findings agree with Clark^33^ and York et al.^21^ and suggest that the combination of rhizosheath and root epidermis should be called the rhizoplane and that it is part of the rhizosphere. The unique environment of the internal roots of plants is called the inner boundary of roots^41^, as was also recognized by York et al.^21^ Our results showed that the bacterial and fungal communities in roots were significantly different from those in the rhizosheath and rhizosphere soil, and although there was no significant difference in fungal community between roots and root epidermis, there were differences in bacterial community. The root system and the rhizosheath are on either side of the root epidermis, which can serve as a compartment to separate the root system from soil. In terms of rhizosphere microbial ecology, Philippot^42^ pointed out that the rhizosphere is the interface between plant roots and soil, and that the rhizosphere environment is complex and dynamic. The interaction between various microorganisms affects plant growth and tolerance to biotic and abiotic stresses. However, we suggest that for plants with a rhizosheath, the outer root epidermis is the interface between the root system and the external soil.

In recent years, research on the structure and function of microbial communities in plant roots and the rhizosphere has gradually increased^43,44,45^. Research has not only focused on characterizing microbial diversity, but has also investigated several different interactions between plants and soil microorganisms and their mechanisms. For example, recent reviews have highlighted that the interaction of underground root soil microorganisms is very important for the growth and health of aboveground plants, discussed the overall view of root soil rhizome microbial interaction realized due to progress in omics and bioinformatics technology, and assessed potential strategies for managing complex rhizosphere interactions to improve crop yield^46–48^. Studies on roots, rhizosheath, soil and microorganisms and the mechanisms of their interaction are rare. The main reasons may include: (1) not all plants have a rhizosheath, so its function may be underestimated, and (2) there is no standard method for separation of the rhizosheath from the rhizoplane. The method used in this study to isolate roots, root epidermis, rhizosheath and rhizosphere soil helped to clarify the relationship between the rhizosheath and root epidermis, and microbial differences in the roots. This study demonstrated that the method of separating the plant rhizosheath used in this study is feasible, and can provide guidance for the extraction of other plant rhizosheath microorganisms and the study of their interactions with plants.

Bergmann et al.^49^ found that the growth of several *Gramineae* plants in low nutrient and low moisture dune environments may be mediated by nitrogen fixing bacteria related to the rhizosheath. In addition, studies have shown that the rhizosheath can improve the drought resistance of plants^14,15^, but whether this is related to the function of some microorganisms in the rhizosheath needs further investigation. It has been shown that there are nitrogen fixing bacteria in the rhizosheath, and nitrogen fixing bacteria can provide ammonia to plants in nitrogen limited soil^50–52^. There are few studies on other functions of microorganisms in root epidermis, which may be due to the limited availability of experimental techniques. Therefore, our next work will be to culture and verify the function of culturable microorganisms in the rhizosheath, root epidermis and rhizosphere soil, and analyze their possible functions by macrogenome sequencing.

## Conclusion

This study provided a feasible method to separate the rhizosheath and root epidermis. This provides the possibility for further study on microorganisms in the rhizosheath and root surface. We suggest that the root epidermis can be seen as a compartment that separates the root system from the soil and that it may act as the interface between the host and the external environment. Microorganisms at this interface potentially have some important functions, which require further study. The differences in composition and function of microorganisms inside and outside the rhizosheath prompt us to reexamine its potentially important role in plant growth. This study has provided a new method and theoretical guidance for further investigation of the function and ecological significance of the rhizosheath.

## Data Availability

The datasets used and/or analyzed during the current study are available from the corresponding author on reasonable request.
